# Modified Vaccinia Virus Ankara Exerts Potent Immune Modulatory Activities in a Murine Model

**DOI:** 10.1371/journal.pone.0011400

**Published:** 2010-06-30

**Authors:** Miriam Nörder, Pablo D. Becker, Ingo Drexler, Claudia Link, Volker Erfle, Carlos A. Guzmán

**Affiliations:** 1 Department of Vaccinology and Applied Microbiology, Helmholtz Centre for Infection Research, Braunschweig, Germany; 2 Institute of Virology, Technische Universität München and Helmholtz Centre Munich, Munich, Germany; 3 Clinical Cooperation Group Antigen Specific Immunomodulation, Technische Universität München and Helmholtz Centre Munich, Munich, Germany; Institut de Pharmacologie et de Biologie Structurale, France

## Abstract

**Background:**

Modified vaccinia virus Ankara (MVA), a highly attenuated strain of vaccinia virus, has been used as vaccine delivery vector in preclinical and clinical studies against infectious diseases and malignancies. Here, we investigated whether an MVA which does not encode any antigen (Ag) could be exploited as adjuvant *per se*.

**Methodology/Principal Findings:**

We showed that dendritic cells infected *in vitro* with non-recombinant (nr) MVA expressed maturation and activation markers and were able to efficiently present exogenously pulsed Ag to T cells. In contrast to the dominant T helper (Th) 1 biased responses elicited against Ags produced by recombinant MVA vectors, the use of nrMVA as adjuvant for the co-administered soluble Ags resulted in a long lasting mixed Th1/Th2 responses.

**Conclusions/Significance:**

These findings open new ways to potentiate and modulate the immune responses to vaccine Ags depending on whether they are co-administered with MVA or encoded by recombinant viruses.

## Introduction

Several poxviral vector systems are under clinical evaluation for vaccine development against infectious diseases and cancer. One of these vectors is based on the highly attenuated modified vaccinia virus Ankara (MVA) that was originally obtained by attenuation over more than 500 passages in chicken embryo fibroblast cultures [1], [2]. During its attenuation, MVA lost ∼15% of its parental genome, including genes that regulate viral host range and evasion of host immune response. The replication in most mammalian cells is extremely limited and in non-permissive human cell lines only immature viral particles are formed [2], [3], [4], [5]. Therefore, dissemination within the host is precluded in most species, including humans. MVA also showed an excellent safety record when administered during the smallpox eradication campaign in approximately 150,000 individuals, including many persons at risk for the conventional pox vaccines [6], [7], [8]. Recombinant MVA (rMVA) expressing immunogens from a variety of infectious agents (*e.g.*, HIV, *Plasmodium falciparum*) or tumor-associated antigens (Ags) have been successfully tested in phase I and II clinical trials [9], [10], [11], [12], [13], [14], [15]. In spite of this, most commercial vaccines are based on formulations encompassing purified Ag(s) and adjuvant(s). Thus, considering the fact that the immune system has evolved to respond to “danger” and that molecules delivering such a “danger” signal(s) (*e.g.*, pathogen associated molecular patterns) are currently been investigated as potential adjuvants [16], and other viral vectors such as influenza virus has been investigated for their adjuvant properties [17], we thought to evaluate whether MVA could be also used as an adjuvant, since it should provide a signal to the innate immune system for its recognition, as occurs for other viruses [18], [19], [20], [21].

Dendritic cells (DCs) are the major link between the innate and the adaptive immune system, and MVA is able to target them *in vivo*, which is a prerequisite in the induction of an adaptive immune response, not only by MVA but also by other poxviruses [22], [23], [24], [25], [26], [27]. However, *in vitro* studies investigating the effect of MVA on DCs showed contrasting results. Behboudi *et al.* reported that rMVA induces DC dysfunction in the murine system with stimulation of MHC class I down-regulation and apoptosis [28], whereas Drillien *et al.* demonstrated moderate activation of human DCs by MVA [29]. On the other hand, Kastenmüller *et al.* carried out an in depth dissection of the characteristics of rMVA infected human DCs, by analyzing their viability and maturation/activation status. Surprisingly, human DCs infected when still immature (iDC) were more prone to apoptosis or necrosis, whereas mature DC (mDC) tolerate infection for longer periods [30]. The iDC were also impaired in their maturation capacity. The expression of activation markers was reduced in the apoptotic/necrotic subpopulation of MVA infected mDC, whereas in living mDC no down-regulation of co-stimulatory molecules was detected [30]. Nevertheless, it is extremely difficult to perform a side-by-side comparison of these reports and draw solid conclusions, since many of the experimental parameters differed (*e.g.*, mouse versus human DCs, bone marrow derived versus splenic DC, MOIs, incubation time, read-outs). Interestingly, despite *in vitro* studies showing either activation or dysfunction, most *in vivo* reports confirm that MVA is able to induce strong immune responses. It was even postulated that the intrinsic modulatory properties of poxviruses can be exploited for the establishment of immune interventions [31]. Nevertheless, little has been done to understand the underlying events to these contrasting reports and only few systematic studies were carried out to investigate the putative immune potentiating activities of MVA [31], [32], [33]. Furthermore, the immune responses evoked after co-administration of an Ag with non-recombinant (nr) MVA as adjuvant have not been yet systematically dissected.

Here, we demonstrate that the differences in the findings in the above mentioned *in vitro* studies are mainly due to the use of different experimental settings, namely, the arbitrary selection of a particular multiplicity of infection (MOI) which might in turn lead to either impaired or enhanced DC function. In contrast to the dominant T helper (Th) 1 responses which have been observed using rMVA expressing target Ags, mice immunized by the intramuscular (i.m.) route with ovalbumin (OVA) co-administered with nrMVA elicited mixed Th1/Th2 responses. Interferon (IFN)-γ producing CD8^+^ T cells and *in vivo* lymphocyte-mediated cytotoxic activity were also preserved when OVA was co-administered with nrMVA. The existence of long term immune responses was shown by the presence of Ag specific B and T cell responses even 3 months after the last immunization.

The results presented in this study suggest that nrMVA could be an attractive tool to potentiate responses against co-administered Ags, particularly taking into consideration its excellent safety profile and the fact that it has been already approved for and used in humans.

## Results

### Activation of murine DCs is dependent on the MOI of nrMVA

**Figure 1:**
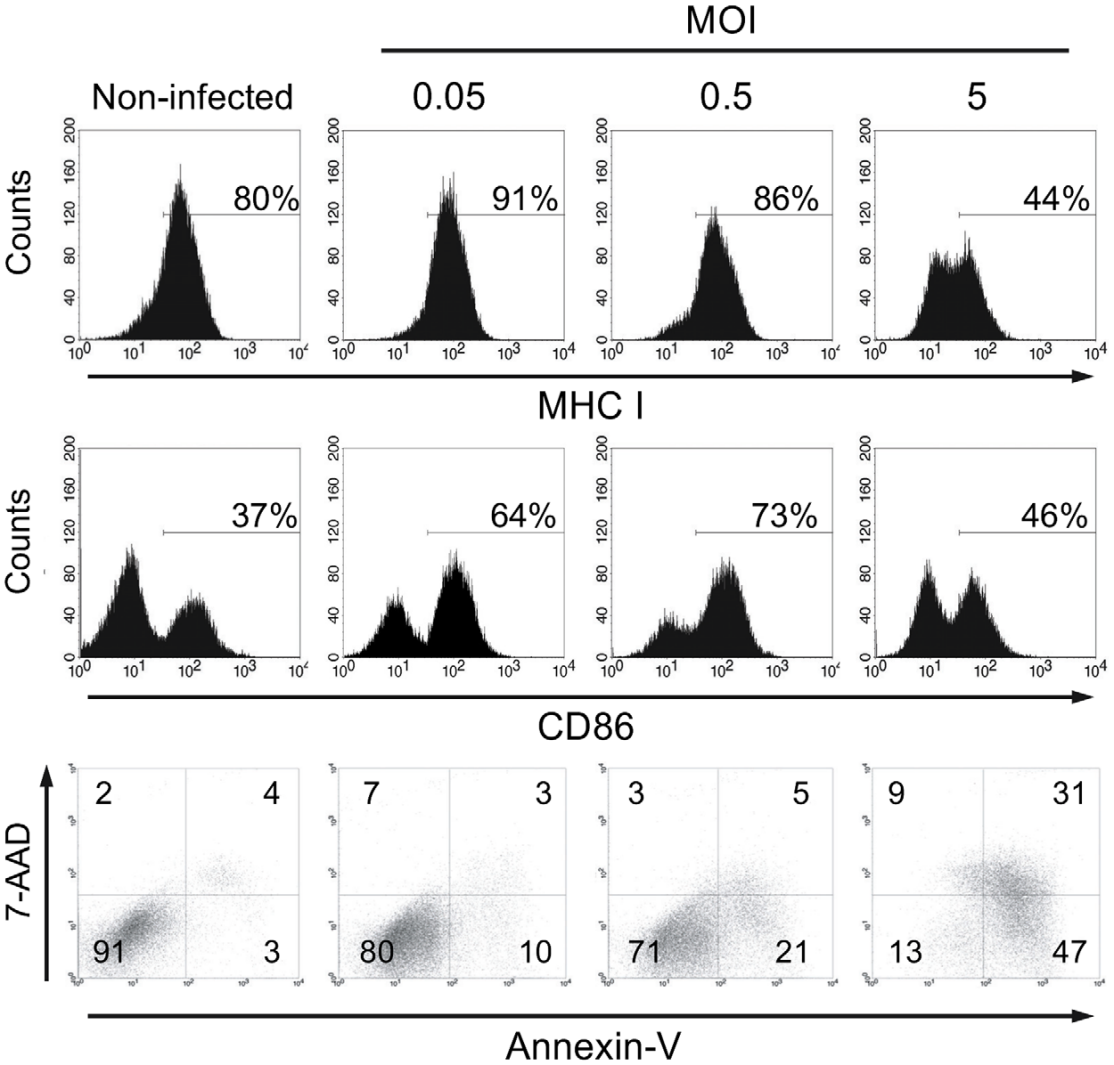
DCs viability and activation depend of the MOI of nrMVA. DCs were infected with nrMVA at different MOIs for 6 h. After washing and further incubation for 16 h, changes in the expression of surface molecules on non-infected and nrMVA-infected DCs were measured by flow cytometry. DC viability was assessed by FACS using Annexin-V (apoptotic cells) and 7-AAD (necrotic cells) after infection with nrMVA at different MOIs. The MOI of 0.05, 0.5 and 5 were arbitrarily considered as representative of low, intermediate and high MOI, respectively. Data are representative of 3 independent experiments.

Murine bone marrow (BM)-DCs were infected with nrMVA at different MOIs (0.05, 0.5 and 5, which were in turn considered as low, intermediate and high, respectively). The DC activation status after infection was evaluated by assessing the expression of the co-stimulatory molecules CD80 and CD86, as well as by their capacity to present MHC class I and II restricted epitopes to OVA-specific CD8^+^ or CD4^+^ T cells, respectively. At all tested MOIs, DCs showed an increase in the expression of CD86 when compared to the non-infected DCs, with the highest expression of CD86 observed in cells infected with the intermediate MOI (MOI 0.5, 73% of CD86^hi^ , Fig. 1). At the highest MOI tested CD86 expression was slightly higher than in non-infected cells (46% vs. 37% of CD86^hi^ , Fig. 1), however, CD86 expression was considerably lower than on DCs infected at the intermediate MOI. As for CD86, CD80 showed the highest expression at intermediate MOI (66%) followed by the lowest MOI (60%), but in contrast to CD86, at the highest MOI tested, CD80 expression was not higher than in non-infected DC (44% and 43% respectively). The MHC class I complex that has a high basal expression on non-infected cells showed an increased expression at low MOI, but its expression inversely decreased with increasing MOI in a dose-dependent manner. At the highest MOI tested, we observed a lower expression of MHC I as compared to DCs infected with the lower MOI (Fig. 1). A similar phenomenon was observed for the expression of the MHC class II complex (data not shown). We have also evaluated the effect of the MOI on cell viability. We observed that the number of apoptotic cells (*i.e.*, Annexin-V^+^) increased with the MOI in a dose-dependent manner. The same holds true for the number of dead cells (*i.e.*, Annexin-V^+^ and 7-AAD^+^). Nevertheless, the number of live cells at low and intermediate MOI was only slightly lower (80% and 71%, respectively) than the non-infected control cells (91%), but there was a remarkable decrease in the number of living DCs after infection at the highest MOI (13%; Fig. 1). Thus, we found that there is a window for the optimal infection of murine BM-DCs *in vitro* in order to achieve the maximum level of activation together with a comparable low number of dead and apoptotic cells (an schematic representation is shown in supplementary Fig. S1). These observations might also explain some of the contrasting results reported in the literature, since different studies were performed using different MOI without carrying out an exhaustive analysis of the optimal dose to be used in the experimental setting to guarantee maximal activation and function of the DCs.

### Improved Ag processing and presentation by DCs infected in vitro at intermediate MOI of nrMVA add-mixed with OVA

**Figure 2:**
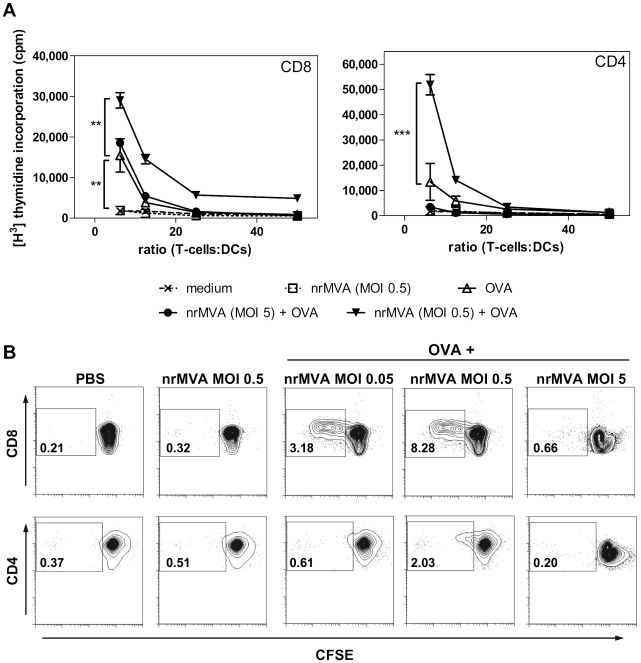
DCs infected with nrMVA stimulate OVA presentation to T cells. DCs were infected for 6 h with nrMVA at a MOI of 0.05, 0.5 or 5 in the presence of OVA protein (10 µg/ml). Non-stimulated and OVA pulsed DCs were used as controls. (*A*) After infection, DCs were cultured with either naïve CD8^+^ T cells from OT-I mice (left panel) or naïve CD4^+^ T cells from OT-II mice (right panel) at different ratios for 5 days. Cells were pulsed with [^3^H]-thymidine 16 h before harvesting. Proliferation of T cells was assessed by measuring [^3^H]-thymidine incorporation. (*B*) MVA-infected DCs were cultured with CFSE labeled naïve T cells of OT-I (upper panel) or OT-II mice (lower panel) for 5 days. Proliferation was assessed by flow cytometry. *p*<0.01 (**); *p*<0.001 (***).

We then evaluated whether the effect of the MOI observed on the activation markers and the viability is reflected in the Ag presentation capacity of DCs. To this end, the capacity of MVA to improve the ability of DCs to process and present OVA to Ag-specific CD8^+^ or CD4^+^ T cells from OT-I or OT-II transgenic mice was evaluated (Fig. 2). DCs pulsed with OVA and infected with nrMVA at different MOIs were washed to eliminate MVA which did not enter the cells. DCs were then further incubated for 16 h, washed and co-cultured with OVA-specific CD4^+^ or CD8^+^ T cells for 5 days. DCs pulsed with OVA and infected with nrMVA at an intermediate MOI (*i.e.*, MOI 0.5) were the most efficient in stimulating both CD4^+^ and CD8^+^ T cells (*p*<0.001 and *p*<0.01, respectively, at T cell:DC ratio of 6.25 as compared with OVA alone, Fig 2). However, when the MOI is further increased to 5, the Ag presentation capacity was significantly impaired (Fig. 2).

### A strong IgG production is triggered by using nrMVA as adjuvant for soluble Ags

**Figure 3:**
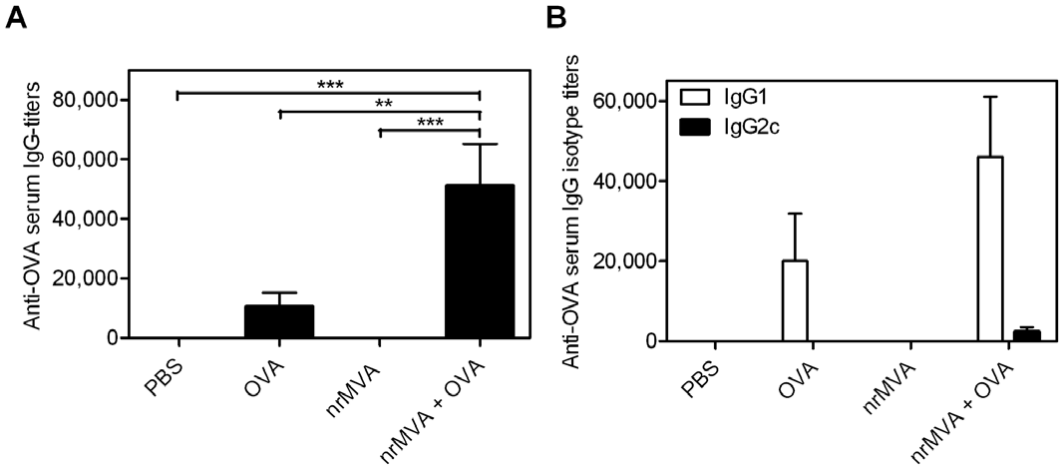
The use of nrMVA as adjuvant triggers OVA-specific humoral responses. (*A*) OVA-specific IgG titers and (*B*) OVA-specific IgG1 and IgG2c isotypes were determined by ELISA. Each bar represents the group mean end-point titer, the SEM is indicated by vertical lines. The results were statistically significant at *p*<0.001 (**); *p*<0.0001 (***).

Next, we evaluated the capacity of nrMVA to act as adjuvant by enhancing the immune responses against co-administered Ags. To this end, the model Ag OVA was co-administered twice with nrMVA to mice by the i.m. route on day 1 and 28. Mice immunized with OVA plus nrMVA showed a strong OVA-specific IgG production in comparison with control mice immunized with either PBS or nrMVA (*p*<0.0001; Fig. 3*A*). Mice immunized with OVA alone showed only a weak OVA-specific IgG response. Of note, it is important to mention that the antibody (Ab) response induced after co-administration of OVA and nrMVA is significantly stronger than the Ab response obtained using rMVA-OVA (*p*<0.0001, Supplementary Fig S2). On the other hand, the subclass distribution of OVA-specific IgG showed that the increment in the IgG levels was mainly due to IgG1 (Fig. 3*B*). Mice immunized with OVA and nrMVA as adjuvant showed a long-lasting Ab response. Due to the fact that the IgG subclass has a half-life of approximately 3 weeks, we decided to investigate the number of Ab-secreting cells (ASCs) in BM, which is known to be responsible for the maintenance of Ab titers over long periods of time [34], [35]. Therefore, 3 months after the last immunization the ability of BM cells to secrete total and OVA-specific IgG after 6 h of re-stimulation was determined by ELISPOT assay. This short incubation period is not enough for the differentiation of memory B cells into ASCs; thus, only actively secreting plasma cells are determined [36]. High numbers of OVA-specific IgG secreting cells were observed in the BM of mice vaccinated with OVA co-administered with nrMVA as adjuvant (Supplementary Fig. S3), showing the long lasting effect of the vaccination.

**Figure 4:**
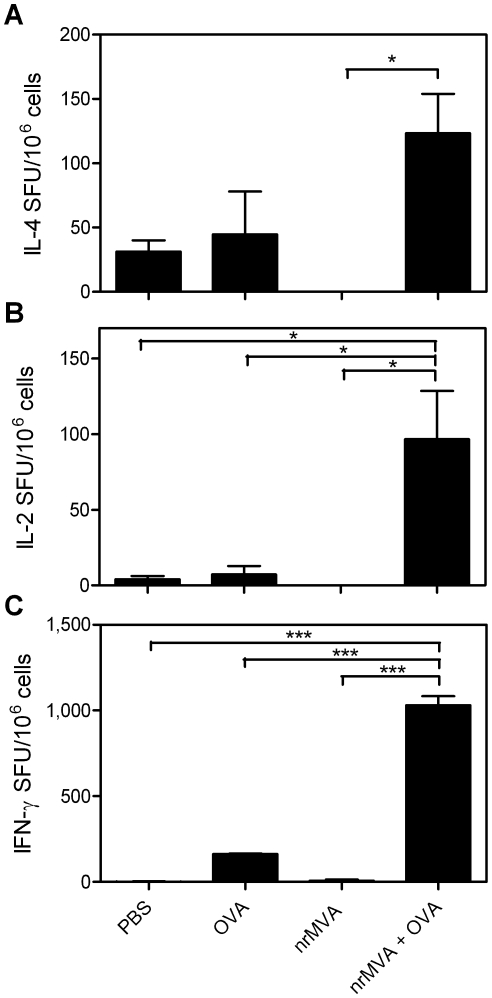
IL-4-, IL-2- and IFN-γ-producing cells were stimulated using nrMVA as adjuvant. Splenocytes of immunized mice were re-stimulated *in vitro* for 48 h with the OVA protein to determine the numbers of (*A*) IL-4- and (*B*) IL-2-secreting cells. (*C*) Cells were also stimulated for 16 h with the dominant MHC class I restricted peptide SIINFEKL to determine the numbers of IFN-γ secreting CD8^+^ T cells. Results are presented as specific spot forming units (SFU)/1×10^6^ cells. The SEM of quadruplicate values is indicated by vertical lines. The values reported are those obtained from stimulated cells subtracted of background from non-stimulated cells. Values of non-stimulated cells were lower than 50 SFU/1×10^6^ cells. The results were statistically significant at *p*<0.05 (*); *p*<0.0001 (***).

We also analyzed the number of interleukin (IL)-4 secreting cells present in the spleens from vaccinated mice after *in vitro* re-stimulation with OVA. Splenocytes from animals immunized with OVA alone did not show any significant difference with respect to the control group, whereas those from mice receiving OVA co-administered with nrMVA showed a significant increment in the number of IL-4 secreting cells (*p*<0.05, Fig. 4*A*). These results correlate with a stimulation of Th2 cells suggested by the IgG isotype analysis.

### Immunizations using nrMVA as adjuvant also stimulate the induction of Ag-specific cytotoxic CD8^+^ T cells

**Figure 5:**
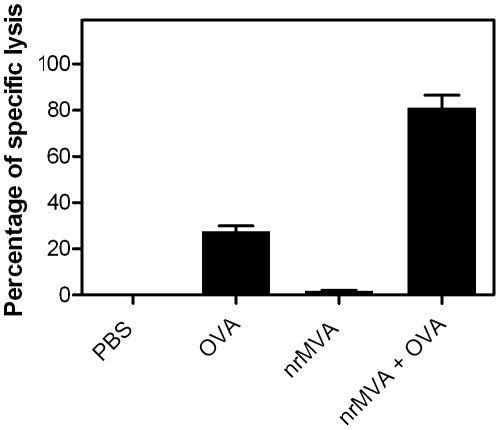
T cell mediated cytotoxic activity was stimulated using nrMVA as adjuvant. CFSE labeled control and target cells pulsed with the OVA peptide (SIINFEKL) were transferred to vaccinated mice. After 16 h, animals were sacrificed and specific lysis was analyzed by flow cytometry. Results correspond to mean values of each group (n = 3) from one representative out of three independent experiments.

The cellular immune responses induced by the immunization using nrMVA as adjuvant were further evaluated by assessing the cytokine production by spleen cells re-stimulated *in vitro* with either OVA protein or peptides. Co-administration of OVA with nrMVA triggered the induction of IL-2 production by splenocytes re-stimulated with OVA (*p*<0.05; Fig. 4*B*). The capacity of CD8^+^ T spleen cells to produce IFN-γ in response to the re-stimulation with a peptide (SIINFEKL) encompassing a MHC class I restricted immunodominant OVA epitope was also significantly increased in mice injected with OVA plus nrMVA respect to those receiving either PBS (*p*<0.0001) or OVA alone (*p*<0.0001), 400- and 6-fold respectively (Fig. 4*C*). The level of IFN-γ was also increased in supernatants from splenocytes derived from mice vaccinated with nrMVA plus OVA respect to those receiving OVA alone after *in vitro* re-stimulation with OVA protein (1199 and 404 pg/ml, respectively). Three months after the last immunization, BM cells were isolated and used to determine whether the observed IFN-γ response was transient [37]. We found that in mice vaccinated with OVA together with nrMVA the number of IFN-γ secreting cells was higher than in mice that received OVA alone (*p*<0.05; data not shown), thereby demonstrating that it was not a transient response, but rather long lasting immunity. The effector function of CD8^+^ T cells was then examined by performing *in vivo* killing assays. One week after the last immunization mice were injected by i.v. route with OVA peptide-pulsed target spleen cells to assess the cytotoxic capacity in vaccinated mice. Control mice immunized with PBS or nrMVA alone showed no cytotoxic activity, whereas those which received OVA alone showed a minimal cytotoxic response. In contrast, mice vaccinated with OVA co-administered with nrMVA showed a high Ag-specific cytotoxic response (80% lysis, *p*<0.01; Fig. 5). This demonstrates that the use of nrMVA as adjuvant also induces a strong CD8^+^ T cell component. In addition, as observed with the IFN-γ response, the cytotoxic activity of CD8^+^ T cells was retained even 3 months after the last immunization in mice vaccinated with nrMVA add-mixed with Ova (64% of lysis; data not shown).

## Discussion

As attenuated virus, MVA possesses the ideal profile to be investigated as adjuvant, since it could provide a signal to the innate immune system for its recognition, as occurs for other viruses [21]. In this study, we focused on investigating the immunomodulatory properties of nrMVA by performing both *in vitro* and *in vivo* studies.

A putative effect of MVA as adjuvant most probably relies on its ability to target DCs [26]. Most of the current knowledge on the interactions of poxviruses with DCs is derived from infection studies of human DCs with the vaccinia virus. In this context, it was reported that infection of monocyte-derived human DCs with the vaccinia virus leads to an inhibition of their maturation, phagocytic capacities and migration. Moreover, their capacity to stimulate T cells *in vitro* seems to be markedly decreased [23], [38], [39]. It was also shown that rMVA infection of DCs leads to apoptosis, MHC class I down-regulation and impairment of DCs ability to induce T cell proliferation [28]. On the other hand, most of these effects are abrogated when gating on living DCs [30]. The dissection of the specific effects triggered by rMVA infection on iDCs and mDCs showed that iDCs are more prone to apoptosis, which in turn leads to impaired function. It was suggested that the lower dose required to infect iDCs might be explained by their higher phagocytic capacities.

To address this issue in a systematic manner, we infected murine derived BM-DCs with nrMVA *in vitro* at different MOI (*i.e*, 0.05 to 5), and assess their capacity to process and present exogenously pulsed OVA as a model Ag. It is important to highlight that in our studies we used nrMVA to assess the adjuvant properties of MVA, whereas most studies reported in the literature used rMVA encoding for different heterologous Ags. This might influence, at least in part, the results obtained due to the fact that protein Ags have different half-life as a result of glycosylation patterns, signal peptides or also due to different expression patterns (*e.g.* membrane-associated versus cytoplasmic protein). Furthermore, some of the expressed Ags might have intrinsic immune modulatory properties.

Our results demonstrate that DC activation after infection with nrMVA tightly depended on the infectious dose used. When DCs were infected *in vitro* with nrMVA at low MOI they became activated and showed the ability to efficiently present OVA-epitopes to Ag-specific naïve T cells, thereby inducing their proliferation. In contrast, when DCs were infected with nrMVA at a high MOI a reduction in the expression of MHC class I molecules was induced with a concomitant increase in the numbers of apoptotic and dead cells. This results in an impaired capacity to present Ags to T cells, which is in turn in agreement with previous studies [28]. Of note, infection with rMVA expressing OVA at high MOI increases only slightly the number of apoptotic cells and does not significantly affect the Ag presentation functions of DCs (data not shown). This suggests that the expression of a heterologous Ag might have a considerable influence on cell viability and the final outcome of the immune response following vaccination. Thus, the data obtained using different rMVAs should not be directly extrapolated to either other rMVAs or to effects observed by using nrMVA as adjuvant.

Our studies show that the infection of DCs with nrMVA at an optimized MOI results not only in a strong activation of the DCs, but also in a remarkable increased ability to act as APC. Concerning the observed class I restricted responses, they can be only explained by cross-presentation (*i.e.*, the Ag is not produced by the cell but given exogenously). However, it is yet to be determined whether it was the result of a direct Ag cross presentation or an “indirect” cross presentation of apoptotic cells which have taken up the Ag. Most probably, both pathways are involved to different extents. Independently of the underlying mechanisms, our *in vivo* studies showed that co-administration of nrMVA resulted in consistently enhanced immune responses against the model Ag OVA. The obtained results demonstrate that in contrast to the strong Th1 responses with low antibodies titers which are reported after administration of rMVA-OVA [40], a mixed Th1/Th2 response pattern was observed using nrMVA as adjuvant. Co-administration of OVA with nrMVA was also able to stimulate an increment in the number of IFN-γ producing cells after re-stimulation with a MHC class I restricted OVA peptide *ex vivo*, as well as a potent *in vivo* killing activity, thereby showing that CD8^+^ effector T cells are also stimulated following immunization. The elicited responses were not transient, as demonstrated by the detection of OVA-specific IFN-γ secreting CD8^+^ T cells and cytotoxic activities 3 months after immunization.

In conclusion, we found that the differences on viability, maturation phenotype and stimulatory potential of MVA-infected DC observed *in vitro* as reported in the literature are most probably due to the individual conditions used to infect DCs. The activation of MVA is MOI dependent, and there is a window in which DC activation and Ag presentation are optimal for the stimulation of T cells. High MOI induced apoptosis and, as other authors proposed, it might increase cross presentation by other non-infected DCs, which in turn could play an important role *in vivo*. In addition, we showed that nrMVA *per se* is able to provide the critical signals to the immune system to induce strong humoral responses against a co-administered Ag *in vivo*, retaining the desired Ag-specific cytotoxic activity usually evoked by rMVA. Thus, considering the urgent need of new adjuvants and the fact that the use of MVA has already demonstrated an impressive safety profile in preclinical and clinical studies, nrMVA appears as a promising candidate adjuvant for vaccine development.

## Methods

### Mice

Six to eight week old-female C57BL/6 mice were purchased from Harlan Winkelmann GmbH (Borchen, Germany) and were treated in accordance with local and European Community guidelines. Mice were kept under specific pathogen-free conditions in individual ventilated cages with food and water *ad libitum*. OT-I mice expressing the OVA_257–264_/K^b^-specific TCR and OT-II mice expressing the OVA_323–339_/A^b^ specific TCR on C57BL/6 background have been described elsewhere [41], [42], [43]. Mice were propagated and maintained in the animal facility at the HZI in Braunschweig. Experiments using animals were done with the permission of the local authorities; permission number: 509.42502/07-04.01, Bezirksregierung Braunschweig.

### Viruses

MVA(II_new_), the vaccinia virus MVA cloned isolate optimized for host range selection, was routinely propagated and titrated by endpoint dilution in chicken embryo fibroblasts to obtain a 50% tissue culture infectious dose [44]. The rMVA-OVA P7.5 was obtained as previously described [27]. For *in vitro* and *in vivo* assays, MVA wild type was purified by ultra centrifugation through a 36% sucrose cushion.

### Ags and peptides

OVA (Sigma, USA) was used as Ag for the *in vitro* and *in vivo* studies. The dominant T cell epitope recognized in context of MHC class I molecules (H-2k^b^), OVA_257–264_ peptide (SIINFEKL), and the T cell epitope recognized in the context of MHC class II (I-A^b^), OVA_323–339_ peptide (ISQAVHAAHAEINEAGR), were synthesized at the HZI (Braunschweig, Germany), as previously described [45], [46].

### BM-derived DC culture and *in vitro* stimulation

BM-derived DCs were prepared from C57BL/6 mice using recombinant murine granulocyte macrophage colony-stimulating factor (GM-CSF; BD Pharmingen), as previously described [47], [48]. On day 6 of culture, cells were counted, mixed and divided into 6-well plates to have 5×10^6^ cells/well. Only cultures that showed more than 70% of CD11c^+^ cells at day 6 were used for experiments. DCs were infected with different concentrations of MVA (MOI 0.05, 0.5 and 5) and/or pulsed with the model Ag OVA (10 µg/ml) for 6 h. Then, DCs were washed 3 times with PBS in order to remove unbound MVA and further incubated for 16 h. Thereafter, expression of DCs surface markers was analyzed by FACS or functional assays were performed.

### Flow cytometric analysis

The DC stimulation was evaluated by surface marker expression by flow cytometry. Briefly, cells were incubated with mouse Fc block (BD Pharmingen) at 4°C for 15 min. Cells were stained with fluorescence-labeled antibodies diluted in FACS buffer (PBS-BSA 1%). FITC-labeled mAb against mouse CD40 (3/23), CD80 (16-10A1), CD86 (GL1), H2-K^b^ (AF6-88.5) and I-A^b^ (A6-120.1), as well as PE- or PE-Cy7-labeled mAb against CD11c (HL3 and N418, respectively) were purchased from BD Pharmingen and eBioscience. After 30 min of incubation at 4°C in the dark, cells were washed twice with FACS buffer. Cells were resuspended, transferred to FACS tubes and kept on ice in the dark until analysis, which was performed on a FACSCalibur or a FACS Canto (BD Bioscience) using BD cell Quest™Pro or FACS Diva software and gating on CD11c^+^ cells. The viability of the stimulated DC was analyzed using “Vybrant^R^ Apoptosis Assay Kit #2”, according to the manufacturer's instructions. Apoptosis and dead of infected DCs were assessed by staining with Annexin V labeled with AlexaFluor488 and 7-AAD, respectively. Analysis of apoptotic/dead cells was done gating on CD11c^+^ cells.

### Proliferation assay

The effect of the different stimuli on Ag processing and presentation by DCs was examined by the proliferation of Ag-specific naïve T cells after co-culture with the differentially pre-treated DCs. This test was performed using naïve T cells from OT-I and OT-II transgenic mice. Spleens from OT-I and OT-II mice were aseptically removed and pooled. Organs were mechanically disaggregated in complete medium (RPMI 1640 supplemented with 10% heat inactivated fetal bovine serum, 100 U/ml of penicillin, 50 µg/ml streptomycin, 1 mM L-glutamine) by gently pressing them through a 100 µm mesh using a syringe plunger. The cell suspension was centrifuged and the pellet was resuspended in ACK buffer to lyse erythrocytes. CD4^+^ and CD8^+^ T cells were isolated using the CD4^+^ or CD8^+^ T cell isolation kit (Miltenyi Biotec) from OT-II and OT-I mice, respectively. For the [^3^H]-thymidine incorporation assay, the pre-treated DCs were added in quadruplicates at different T cells:DCs ratios (ranging from 6.25 to 50) to flat-bottom 96-well culture plates. As positive controls, the DCs stimulated with either the corresponding OVA peptides (0.1 µg/ml) or the OVA protein (10 µg/ml) were used. Then, the co-cultured cells were incubated at 37°C with 5% CO_2_ in a humidified atmosphere for 4 days. Before harvesting, the cells were pulsed with [^3^H]-thymidine (1 µCi/well) for 16–18 h. The [^3^H]-thymidine incorporated into the DNA of proliferating cells was determined by a γ-scintillation counter (MicroBeta Trilux, Wallac). Results are expressed as the arithmetic mean of [^3^H]-thymidine uptake in cpm. For the CFSE proliferation assay, T cells were labeled with 1 µM CFSE (Molecular Probes). Labeling was stopped by adding fetal bovine serum. After one wash in complete medium, T cells were adjusted to 1×10^6^ cells/ml and 100 µl of the cell suspension was co-cultured with pretreated DCs in 96-well plates. Cell suspensions were seeded in round-bottom 96-well cell culture plate (TPP, Switzerland). As positive control, T cells were stimulated with Con A, as negative control T cells were cultured without stimulus in complete medium. Plates were incubated for 5 days at 37°C with 5% CO_2_. On day 5, CD4^+^ and CD8^+^ T cells were stained with PE-Cy7 labeled CD4 (RM4-5, eBioscience) and APC labeled CD8 (53–67, eBioscience) Abs, respectively. Proliferation was measured by flow cytometry using a LSR-II FACS machine (BD Bioscience) and analyzed using FACS Diva Software (BD Bioscience).

### Immunizations

Groups of six C57BL/6 mice were immunized on day 1 and boosted on day 28 by i.m. route (biceps femoris) using a 25G needle. Per dose, 100 µl/mouse were injected containing either 50 µg of OVA alone or OVA add-mixed with 1×10^8^ nrMVA as adjuvant. Negative controls received PBS. Serum samples were collected from blood of the tail vein 1 day before each immunization and 1 week after the last immunization, when the mice were killed by CO_2_ inhalation. Sera were stored at −20°C prior to determination of Ag-specific Abs.

### Detection of Ag-specific Abs

OVA-specific Abs were determined by ELISA, as previously described [49]. Briefly, 96-well Immuno MaxiSorp plates (Nunc, Roskilde, Denmark) were coated overnight at 4°C with 100 µl of OVA with a concentration of 5 µg/ml in carbonate buffer (pH 9.6). Then, the wells were blocked, and plates were washed and further incubated with 100 µl of serial two-fold dilutions of sera at 37°C for 1 h. After four washes, the biotinylated γ-chain specific goat anti-mouse detection Ab (Sigma Chemie, Deisenhofen, Germany) was added. The plates were further incubated at 37°C for 1 h. After the plates were washed, peroxidase-conjugated streptavidin (PharMingen) was added and the plates were incubated at 37°C for 45 min. After another four washes, the reaction was developed using ABTS in 0.1 M citrate-phosphate buffer (pH 4.35) containing 0.01% H_2_O_2_, and the absorbance was read at a wavelength of 405 nm. The IgG isotypes present in the serum samples were determined by an ELISA using as secondary Abs biotin-conjugated rat anti-mouse IgG1 and IgG2c (Southern Biotechnology Associates, Birmingham, AL). The absorbance values were plotted against the dilutions, and the endpoint titers were determined as the highest sample dilution that gave an A_405_ of ≥0.2 above the background values. The results were expressed as mean±SEM for each group.

### Determination of IFN-γ in supernatants of re-stimulated splenocytes

To quantify the IFN-γ secreted by splenocytes re-stimulated *in vitro* using OVA protein, supernatants were collected on day 4 and stored at −70°C until processing. Then, the concentration of IFN-γ was measured using the mouse Th1/Th2 BD Cytokine Bead Array (BD Bioscience), according to the manufacturer's instructions.

### Detection of IFN-γ-, IL-4- and IL-2-producing cells

The numbers of IFN-γ-, IL-4-, and IL-2-secreting cells in the spleen and BM of immunized mice were determined by ELISPOT assay, according to the manufacturer's instructions (Becton Dickinson). Briefly, polyvinylidene difluoride plates (Millipore, Bedford, MA) were coated with anti-IFN-γ or anti-IL-2 or anti-IL-4 specific capture antibodies (100 µl/well). After washing and blocking, cells (1×10^6^ cells/well) were incubated in triplicate in the absence or presence of MHC class I restricted OVA peptide (for IFN-γ) or OVA (for IL-4 and IL-2) for 16 h and 48 h, respectively. Then, plates were washed and 100 µl of the corresponding biotinylated detection antibody was added to each well, and plates were incubated for 1 h at room temperature. After several washes, the plates were further incubated for 1 h with 100 µl/well of peroxidase-conjugated streptavidin (BD Pharmingen, Germany). The spots were developed by using 3-amino-9-ethylcarbazole (Sigma) in 0.1 M acetate buffer (pH 5.0) and 0.05% H_2_O_2_. The reaction was stopped by rinsing the plates with tap water, and the plates were air-dried. The spots were scanned with a CTL ELISPOT reader (ImmunoSpot series 3A) and were counted by using ImmunoSpot image analyzer software v3.2.

### Detection of IgG-secreting cells

The numbers of total and Ag-specific IgG secreting cells in the BM of immunized mice were determined by ELISPOT assay. Briefly, polyvinylidene difluoride plates (Millipore, Bedford, MA) were coated with either 100 µl/well of anti-IgG capture Ab (Sigma, Germany) or OVA (5 µg/ml) to determine total and Ag-specific Abs-secreting cells, respectively. Different concentrations of BM cells were incubated in quadruplicate for 6 h. Then, the plates were washed, 100 µl of the biotinylated detection Ab (anti-IgG, Sigma) was added to each well, and the plates were incubated at 4°C overnight. After several washes, the plates were incubated for 1 h with 100 µl/well of peroxidase-conjugated streptavidin (BD Pharmingen, Germany) and then processed as described above.

### *In vivo* killing assay

Suspensions of splenocytes from naïve C57BL/6 mice were depleted of red blood cells and split into two equal portions. One portion was labeled with a high concentration (1 µM) of CFSE (CFSE^hi^; Molecular Probes) and incubated at 37°C for 1 h with the dominant OVA peptide (amino acids 257 to 264) at a concentration of 15 µg/ml. The other portion was labeled with a low concentration (0.1 µM) of CFSE (CFSE^lo^) and was further incubated at 37°C for 1 h without peptide. Equal numbers of each cell population were mixed. A total amount of 2×10^7^ cells was adoptively transferred by i.v. injection into the immunized mice. Cells from the spleen were analyzed by flow cytometry after 16 h with a FACSCalibur interfaced with BD Cell Quest Pro software. The percentage of OVA-specific lysis was determined by the loss of the peptide-pulsed CFSE^hi^ population and compared to the control CFSE^lo^ population. The following formula was used to calculate the percentage of specific lysis: 100−{[(% CFSE^hi^ in immunized mice/% CFSE^lo^ in immunized mice)/(% CFSE^hi^ in control mice/% of CFSE^lo^ in control mice)]×100}.

### Statistical analysis

Statistical analysis was performed using the software GraphPad Prism version 5.02 for Windows. The significance of the differences observed between three or more groups was determined using the one-way ANOVA followed by the Turkey-Kramer post test. Differences were considered significant at *p*<0.05.

## Supporting Information

Figure S1 Schematic representation of the viability and activation levels of non-infected and nrMVA-infected DCs at different MOIs. The MOI of 0.05, 0.5 and 5 were arbitrarily considered as representative of low, intermediate and high MOI, respectively.
(0.15 MB TIF)

Figure S2 Comparison of immune responses induced by OVA using nrMVA as adjuvant and rMVA-OVA P7.5. (A) OVA-specific serum IgG titers. Each bar represents the group mean end-point titer, the SEM is indicated by vertical lines. (B) OVA specific IFN-γ secreting CD8^+^ T cells. Splenocytes of immunized mice were re-stimulated *in vitro* for 16 h with the dominant MHC class I restricted peptide SIINFEKL. Results are presented as specific spot forming units (SFU)/1×10^6^ cells. The SEM of quadruplicate values is indicated by vertical lines. The values reported are those obtained from stimulated cells subtracted of background from non-stimulated cells. Values of non-stimulated cells were lower than 50 SFU/1×10^6^ cells. The results were statistically significant at *p*<0.001 (**); *p*<0.0001 (***).
(0.89 MB TIF)

Figure S3 Long lasting IgG specific are maintained by BM Ab-secreting cells in mice immunized with OVA and nrMVA. BM total IgG secreting cells or BM OVA-specific IgG secreting cells from mice vaccinated with nrMVA alone or OVA alone or OVA add-mixed with nrMVA were measured by ELISPOT. One representative ELISPOT well from each group is shown. The total number of spots is indicated in the upper left quadrant of each panel.
(2.75 MB TIF)
