# Supplementary figures and images for: Modified Vaccinia Virus Ankara Exerts Potent Immune Modulatory Activities in a Murine Model
Source: PLoS One. 2010 Jun 30;5(6):e11400. doi: 10.1371/journal.pone.0011400 (PMC2900180; doi:10.1371/journal.pone.0011400)

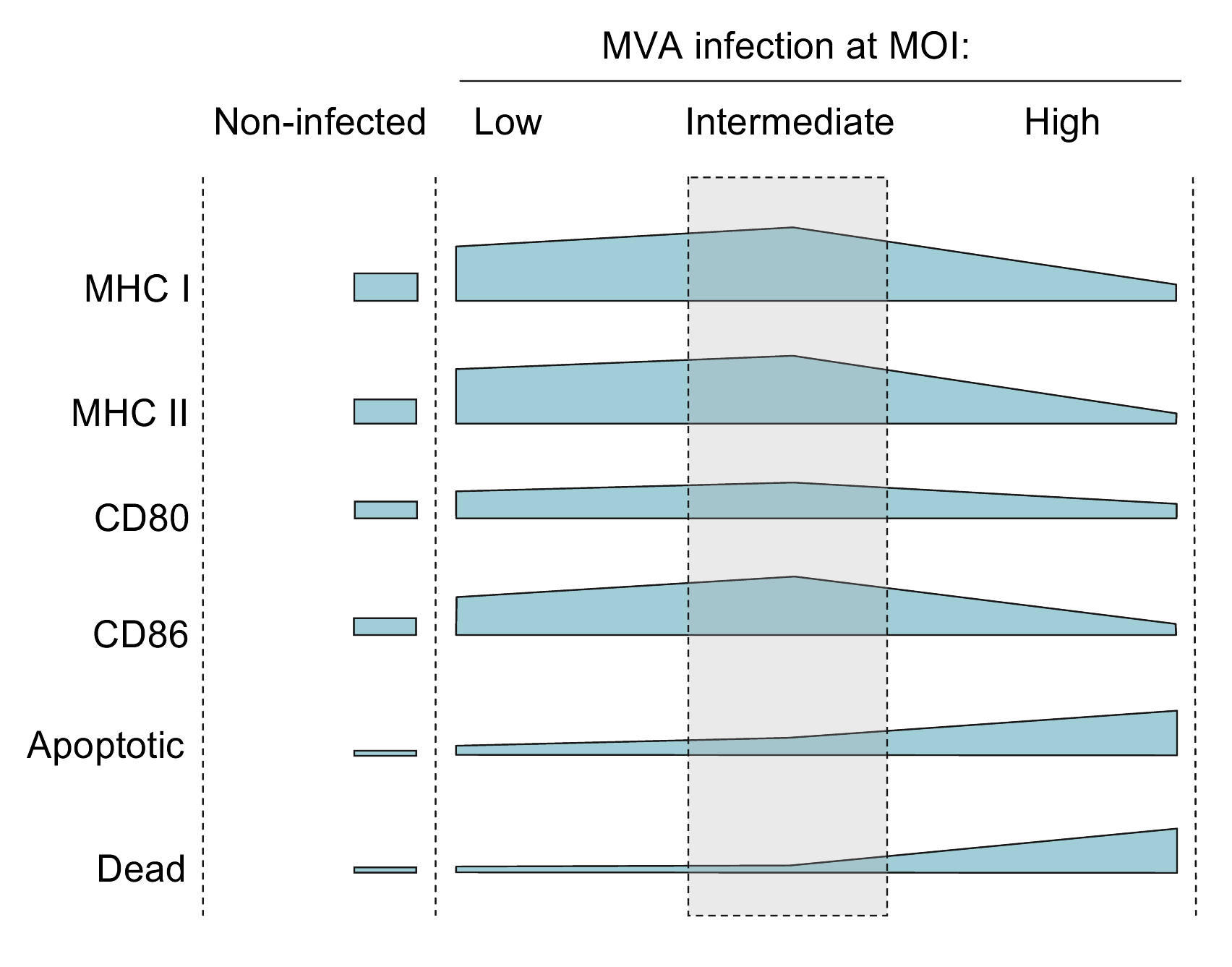

Supplement: Figure S1 — Schematic representation of the viability and activation levels of non-infected and nrMVA-infected DCs at different MOIs. The MOI of 0.05, 0.5 and 5 were arbitrarily considered as representative of low, intermediate and high MOI, respectively. (0.15 MB TIF) [file pone.0011400.s001.tif]

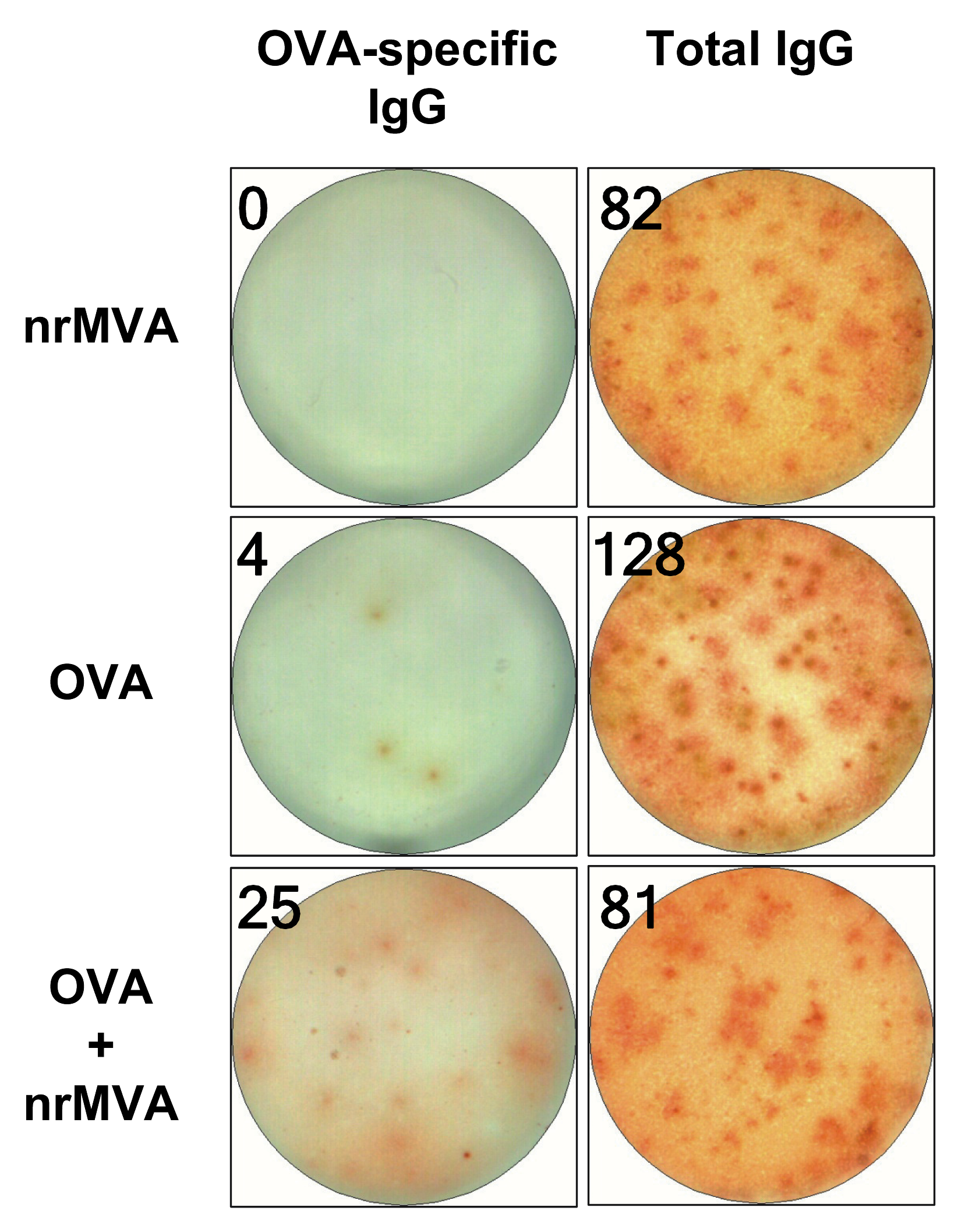

Supplement: Figure S3 — Long lasting IgG specific are maintained by BM Ab-secreting cells in mice immunized with OVA and nrMVA. BM total IgG secreting cells or BM OVA-specific IgG secreting cells from mice vaccinated with nrMVA alone or OVA alone or OVA add-mixed with nrMVA were measured by ELISPOT. One representative ELISPOT well from each group is shown. The total number of spots is indicated in the upper left quadrant of each panel. (2.75 MB TIF) [file pone.0011400.s003.tif]
